# P01-019 – Anti-CCP antibodies are not associated with FMF

**DOI:** 10.1186/1546-0096-11-S1-A23

**Published:** 2013-11-08

**Authors:** H Onur, H Aral, V Arıca, G Bercem, M Usta, Ö Kasapçopur

**Affiliations:** 1 Department of Pediatrics, Istanbul Training and Research Hospital, Istanbul, Turkey; 2 Department of Biochemistry, Istanbul Training and Research Hospital, Istanbul, Turkey; 3 Department of Pediatrics, Mustafa Kemal University Medical Faculty, Hatay, Turkey; 4 Department of Biochemistry, Giresun University Medical Faculty, Giresun, Turkey; 5 Pediatric Rheumatology, Istanbul University, Cerrahpasa Medical Faculty, Istanbul, Turkey

## Introduction

*Familial Mediterranean Fever* (*FMF*) is an autosomal recessive disease that is prevalent among eastern Mediterranean populations, mainly non-Ashkenazi Jews, Armenians, Turks, and Arabs. Arthritis seen in FMF patients is generally acute monoarthritis which predominantly affecting the lower limbs, and it occurs during attack periods and also is a common clinical manifestation in patients with FMF alike Rheumatoid arthritis (RA). *Anti-cyclic citrullinated peptide* (*anti-CCP*) *antibodies* testing is useful in the diagnosis of Rheumatoid arthritis with high specificity. The citrulline residues are essential part of the antigenic determinants recognized by the RA antibodies.

## Objectives

The aim of the study was to show the presence of anti-cyclic citrullinated peptide (anti-CCP) antibodies in child individuals diagnosed with Familial Mediterranean Fever (FMF) .

## Methods

The study group was comprised of one hundred and twenty six patients diagnosed with FMF (female/male (n):66/60); and fifthy healthy control (female/male(n):25/25) . Clinical and laboratory assessments of the FMF patients were performed during attack-free periods. Erythrocyte sedimentation rate (ESR), serum C-reactive protein (CRP), fibrinogen and anti-CCP antibody levels were measured.

## Results

Anti-CCP results were negative in healthy controls and also in all FMF patients. There was not a significant difference in anti-CCP between the patient and the control groups. The patient individuals were divided into four groups according to genetic mutation analysis. The groups has been comprised as M694V/M694V(n=26), M694V/Other(n=38), Other/Other(n=46),Negative(n=16). No significant difference detected between four mutation groups and anti-CCP levels. Our study has shown moderate positive correlations between age (r_s_= 0.271; p= 0.0020), duration of illness (r_s_= 0.331; p<0.0001), colchicinetherapy (r_s_= 0.259; p= 0.004) and anti-CCP levels. Also poor positive correlations between fibrinogen and anti ccp levels was detected (r_s_= 0.192; p= 0.0330). Anti-CCP levels has not shown significance between patients with or without arthritis(p=0.148).

## Conclusion

In conclusion, no published data in children establish anti-CCP values in patients with FMF compared with healthy controls. Our data show that anti-CCP antibodies are not associated with FMF. Anti-CCP does not have a priority for identifying FMF arthritis from the other inflammatory arthritis.

## Disclosure of interest

None declared.

